# Research progress on the role of PTEN deletion or mutation in the immune microenvironment of glioblastoma

**DOI:** 10.3389/fonc.2024.1409519

**Published:** 2024-08-14

**Authors:** Leiya Du, Qian Zhang, Yi Li, Ting Li, Qingshan Deng, Yuming Jia, Kaijian Lei, Daohong Kan, Fang Xie, Shenglan Huang

**Affiliations:** ^1^ Department of Oncology, The Second People’s Hospital of Yibin, Yibin, Sichuan, China; ^2^ Department of Neurosurgery, The Second People’s Hospital of Yibin, Yibin, Sichuan, China; ^3^ Department of Burn and Plastic Surgery, The Second People’s Hospital of Yibin, Yibin, Sichuan, China

**Keywords:** glioblastoma, PTEN, immunity, tumor microenvironment, immunosuppressive

## Abstract

Recent advances in immunotherapy represent a breakthrough in solid tumor treatment but the existing data indicate that immunotherapy is not effective in improving the survival time of patients with glioblastoma. The tumor microenvironment (TME) exerts a series of inhibitory effects on immune effector cells, which limits the clinical application of immunotherapy. Growing evidence shows that phosphate and tension homology deleted on chromosome ten (PTEN) plays an essential role in TME immunosuppression of glioblastoma. Emerging evidence also indicates that targeting PTEN can improve the anti-tumor immunity in TME and enhance the immunotherapy effect, highlighting the potential of PTEN as a promising therapeutic target. This review summarizes the function and specific upstream and downstream targets of PTEN-associated immune cells in glioblastoma TME, providing potential drug targets and therapeutic options for glioblastoma.

## 1 Introduction

Glioma is the most common primary malignant tumor of the central nervous system (1). Its pathological types and molecular characteristics are varied, and about 80% of cases manifest as glioblastoma (GBM). Primary glioblastoma is the brain tumor with the highest degree of intracranial malignancy, characterized by strong invasion and poor prognosis; the average survival time of GBM patients is only 15 months (2, 3). Currently, postoperative adjuvant chemoradiotherapy is the standard treatment for glioblastoma (GBM) but only provides limited survival benefit. Immunotherapy, represented by immune checkpoint inhibitors, has revolutionized the treatment paradigm for many solid tumors, but only a small percentage of GBM patients have shown objective efficacy (4). Compared with other tumors, GBM demonstrates stronger heterogeneity, lower tumor mutation load, and a highly immunosuppressive microenvironment. Due to the strong immunosuppressive tumor microenvironment (TME) of GBM, the application of immunotherapy in GBM remains suboptimal and requires further research (5). The most significant feature of the GBM tumor immune microenvironment is the absence of tumor-infiltrating lymphocytes (TILs) and natural killer cells (NK cells), as well as the elevated levels of tumor-associated macrophages (TAMs), myelogenic suppressor cells (MDSCs) and regulatory T cells (Tregs) (6). Enhancing the immune system’s targeting effect on GBM has emerged as a promising approach to treating tumors.

Phosphate and tension homology deleted on chromosome ten (PTEN) is the first tumor suppressor gene with protein phosphatase activity and lipid phosphatase activity discovered so far. It is located on human chromosome 10q23.3 and regulates a variety of signaling pathways through its bispecific phosphatase activity, thereby regulating the life process of various cells (7). PTEN can be involved in cell cycle regulation, inhibition of tumor cell proliferation, adhesion, metastasis, angiogenesis, and promotion of cell apoptosis, differentiation, senescence, and other physiological and pathological activities. PTEN plays a crucial role in the occurrence and development of a variety of tumors (breast, melanoma, glioblastoma, prostate, liver, lung), and even a slight decrease in PTEN enzyme activity can affect cancer susceptibility (8). Mutations in IDH, PTEN, 1p/19g, TERT, ATRX, BRAF, and H3F3A in gliomas are of great significance for patient prediction and prognosis (**Table 1**) (9, 10). Overall, 40% of GBM cases exhibit PTEN mutation or deficiency, which is associated with a poorer prognosis than PTEN non-deletion GBM (11). Many recent studies have shown that PTEN mediates multiple mechanisms of immunosuppression in GBM immune regulation, and targeting PTEN can enhance the immune response of GBM (12, 13). This study summarizes the direct and indirect effects of PTEN on the various pathways of immune response in GBM, the mechanisms of mutual regulation between PTEN and immune cells in the immunosuppressive microenvironment, and the latest immunotherapy strategies for glioblastoma.

**Table 1:** The mutations genes in GBM patients.

Mutation genes	Location	Function	Clinical trial drugs
IDH	2q33;15q26	Mutated IDH has a gain of function to produce 2-hydroxyglutarate by NADPH-dependent reduction of alpha-ketoglutarate	Ivosidenib
PTEN	10q23.3	PTEN can be involved in cell cycle regulation, inhibition of tumor cell proliferation, adhesion, metastasis, angiogenesis, and promotion of cell apoptosis, differentiation, senescence, and other physiological and pathological activities	
1p/19q	1p/19q	Heterozygous deletions are important in determining the prognosis of glioma patients	
TERT	5p15.33	The TERT is an important component and functional unit of telomerase, which plays a key regulatory role in tumorigenesis and malignant proliferation, among others	
ATRX	Xq21.1	ATRX forms the ATRX-DAXX complex by binding to death structural domain-associated protein (DAXX), which accelerates the process of histone deposition and is involved in the regulation of remodeling chromatin, all of which are of considerable value for the maintenance of the stability of the human genome	
BRAF	7q34	BRAF is a serine/threonine kinase that functions in the MAPKs signaling pathway and is involved as a proto-oncogene in the development of many cancers, including gliomas	Vemurafenib; Dabrafenib

## 2 PTEN is involved in the GBM immunosuppressive pathway

**Figure 1:**
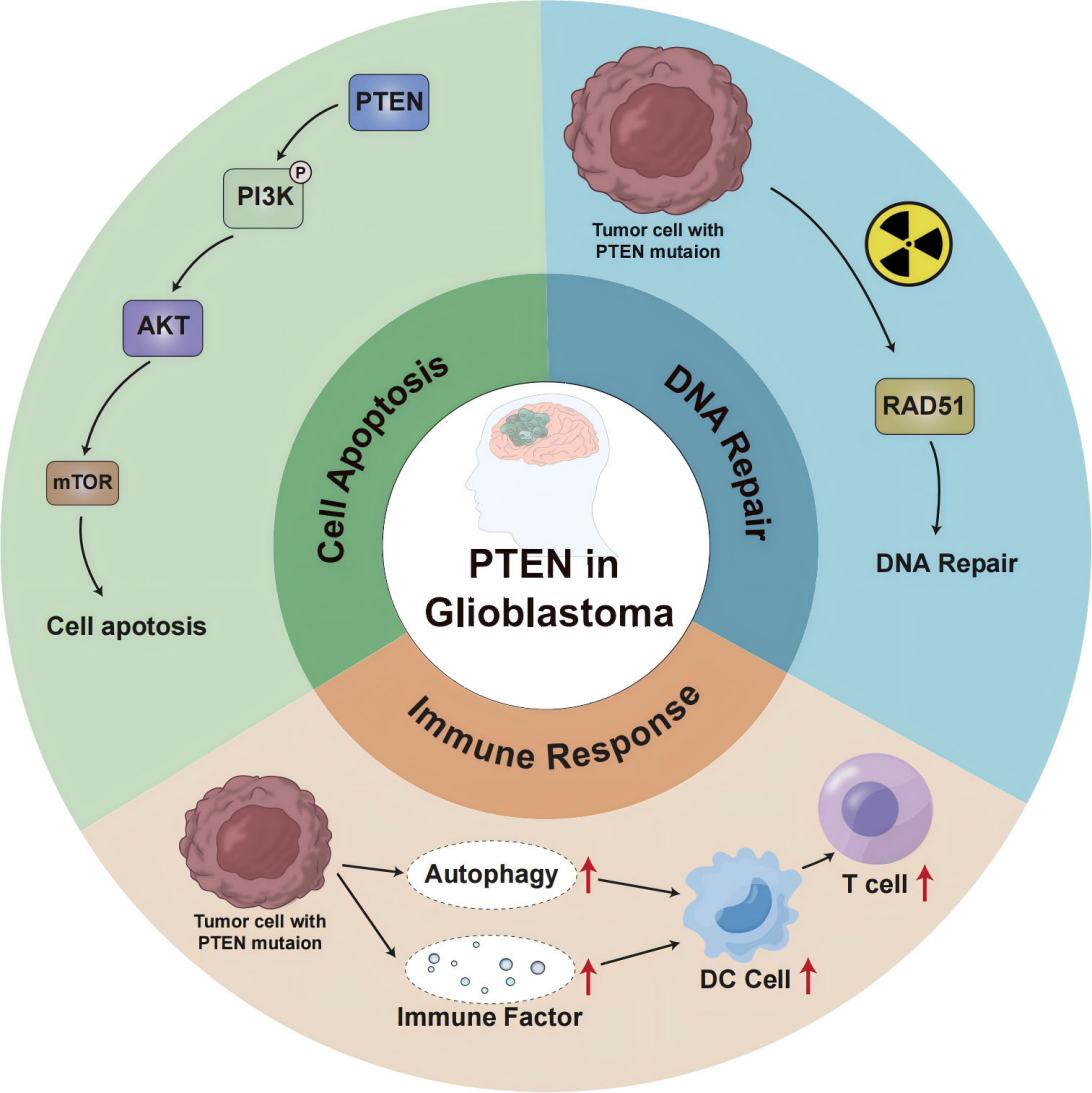
PTEN-mediated signaling pathway and molecular mechanism in GBM.

In glioblastoma, PTEN deletion or mutation may affect the genomic stability, autophagy, and other aspects of the immune response, leading to immunotherapy failure (**Figure 1**). The P13K/Akt/mTOR signaling pathway mediates important physiological functions by regulating the cell cycle, protein synthesis, cell energy metabolism, and other pathways, and plays a central regulatory role in the process of cell proliferation, growth, and differentiation. Moreover, activation of this signal transduction pathway promotes cell survival and proliferation and participates in angiogenesis, thereby promoting tumor formation, tumor invasion, and metastasis (14). Studies (15) suggest that the P13K/Akt/mTOR signaling pathway also plays a key role in the occurrence and development of cerebral glioblastoma. The regulation of PTEN and mTOR plays an essential role in this transduction pathway. The protein encoded by the PTEN gene has phosphatase activity and can negatively regulate the P13K/Akt/mTOR signal transduction pathway by catalyzing the dephosphorylation of 3,4,5 phosphatidylinositol to 4,5 monophosphatidylinositol, thereby inducing cell apoptosis (16). As the upstream site of the P13K/Akt/mTOR signaling pathway, the PTEN gene inhibits tumor formation through negative regulation of this signaling pathway, whereas inactivating the PTEN gene reduces the negative regulation of this pathway and causes malignant changes in cells. Research (17) has shown that PTEN is involved in the tumor immune response, and PTEN deficiency activates the phosphatidylinositol 3-kinase (PI3K-AKT) pathway to form an immunosuppressive microenvironment. The combination of PI3K inhibitor and PD-1 blocker was found to have a synergistic effect in PTEN-deficient tumors and can improve patient prognosis. Furthermore, the PI3K-AKT-mTOR pathway can directly affect the immune response in PTEN-deficient glioblastoma TME (18). Increased PD-L1 cell surface expression induced by PTEN loss led to decreased T-cell proliferation and increased apoptosis. Because PTEN loss is one mechanism regulating PD-L1 expression, agents targeting the PI3K pathway may increase the antitumor adaptive immune responses (19). PIK3CA-mutated PTEN-lost tumors showed a higher prevalence of CD274-positivity than PIK3CA-wild-type PTEN-lost tumors and PTEN-expressed tumors. These findings support the role of PI3K signaling in the CD274/PDCD1 pathway (20). AKT-mediated β-catenin S552 phosphorylation and nuclear β-catenin are positively correlated with PD-L1 expression and inversely correlated with the tumor infiltration of CD8^+^ T cells in human glioblastoma specimens, highlighting the clinical significance of β-catenin activation in tumor immune evasion (21).

In addition to cytoplasmic functions that regulate cell growth and proliferation, PTEN also regulates genomic integrity and the stability of DNA repair in the nucleus. Studies (22) have shown that mice with PTEN deletion tumors exhibit increased genomic and chromosomal instability, resulting in centromeric breaks, chromosomal translocations, and spontaneous DNA double-strand breaks that occur independently of the PI3K-AKT-mTOR pathway. About 40% of GBM cases show a deficiency or mutation of the PTEN gene, which influences neurogenesis and gliogenesis, resulting in increased DNA damage repair and malignant progression of brain tumors (23). In glioblastoma (24), after cell exposure to ionizing radiation, DNA repair is weakened when nuclear PTEN is phosphorylated at position 240. Phosphorylated PTEN binds to chromatin and recruits RAD51 to facilitate DNA repair (25). Due to PTEN inactivation promoting higher genomic instability (26, 27), PTEN-deficient tumors are generally considered pro-inflammatory, exhibiting a greater mutation burden and higher immunogenicity in the TME. To counteract the effects of neoantigens, tumors with highly unstable genomes are likely to be able to suppress the host immune response against pro-inflammatory activity (28).

The expression of PTEN can induce autophagy, while the loss of PTEN function down-regulates autophagy, effectively supporting the development of tumors (29, 30). The etiology and pathogenesis of GBM remain incompletely understood, but growing evidence indicates the involvement of the ubiquitin-proteasome system (UPS) and autophagy-lysosome pathway (ALP) in the occurrence, development, and drug resistance of GBM. These effects are carried out by regulating the degradation of cancer-promoting/cancer-suppressing factors and mediating endoplasmic reticulum stress tolerance and misfolded protein reaction (31, 32). PTEN is frequently mutated in glioblastoma, and ectopic expression of functional PTEN in glioma cells induces autophagy flux and lysosomal mass. Furthermore, proteasome activity and protein ubiquitination are inhibited, restricting tumor development. Interestingly, these effects were independent of PTEN lipid phosphatase activity and the PI3K/AKT/mTOR signaling pathway (33). These findings suggest a novel mTOR-independent signaling pathway through which PTEN can act on intracellular protein degradation, regulating autophagy. In addition, studies reported that the activation of the PI3K/Akt/mTOR-mediated signaling pathway can also inhibit autophagy (34–36). Therefore, the molecular components of the proteolytic system regulated by PTEN could represent an innovative therapeutic target for cancer treatment. Moreover, proteasome inhibitors were found to induce cell death in PTEN-deficient GBM organoids and inhibit tumor growth in mice (37). Proteasome inhibitors could be used as targeted therapies for GBM. Mechanistically, PTEN-deficient GBM cells secrete high levels of galectin-9 (Gal-9) via the AKT-GSK3β-IRF1 pathway. The secreted Gal-9 drives macrophage M2 polarization by activating its receptor Tim-3 and downstream pathways in macrophages. These macrophages, in turn, secrete VEGFA to stimulate angiogenesis and support glioma growth (38). Therefore, this study suggests that blockade of Gal-9/Tim-3 signaling is effective to impair glioma progression by inhibiting macrophage M2 polarization, specifically for PTEN-null GBM. PI3Kβ inactivation in the PTEN- null setting led to reduced STAT3 signaling and increased the expression of immune stimulatory molecules, thereby promoting anti-tumor immune responses (39). These findings demonstrate a molecular mechanism linking PTEN loss and STAT3 activation in cancer and suggest that PI3Kβ controls immune escape in PTEN-mutation tumors, providing a rationale for combining PI3Kβ inhibitors with immunotherapy. NF-κB activation was necessary and sufficient for inhibition of PTEN expression. The promoter, RNA, and protein levels of PTEN are down-regulated by NF-κB. The mechanism underlying suppression of PTEN expression by NF-κB was independent of p65 DNA binding or transcription function and involved sequestration of limiting pools of transcriptional coactivators CBP/p300 by p65. Restoration of PTEN expression inhibited NF-κB transcriptional activity and augmented TNF-induced apoptosis, indicating a negative regulatory loop involving PTEN and NF-κB. PTEN is, thus, a novel target whose suppression is critical for antiapoptosis by NF-κB (40).

In the context of tumor cell death, autophagy may lead to the secretion of damage-related molecular chaperones (41, 42). In addition, dead cancer cells may also release autophagosomes containing multiple tumor antigens, which subsequently induce the maturation of dendritic cells (DCS) and cross-present to T cells, promoting tumor immunity (43, 44). PTEN inhibits autophagy, which hinders an effective anti-tumor immune response. Research (45, 46) has revealed that the biology of the immune system determines the occurrence and progression of tumors through a balance between the effects of autophagy regulation and the tolerance response. Autophagy affects the biological functions of various cell types of the immune system, including natural killer cells, dendritic cells, macrophages, and T and B lymphocytes. Autophagy also regulates the secretion of cytokines and antibodies, which in turn impact the autophagy process itself. Transforming growth factor-β, interferon-gamma-γ, and several interleukins (IL) promote autophagy, whereas IL-4, IL-10, and IL-13 are inhibitors (47). Autophagy can be stimulated by innate immune receptors such as toll-like receptors (48); in adaptive immunity, it is a determinant of antigen presentation, lymphocyte differentiation, and cytokine secretion with tumor suppressor activity (49). Therefore, the ideal treatment combination could involve the combination of existing treatment strategies and autophagy-based inducers (PTEN inducers) to trigger cancer cell death and patient response.

## 3 PTEN affects the GBM immune microenvironment

**Figure 2:**
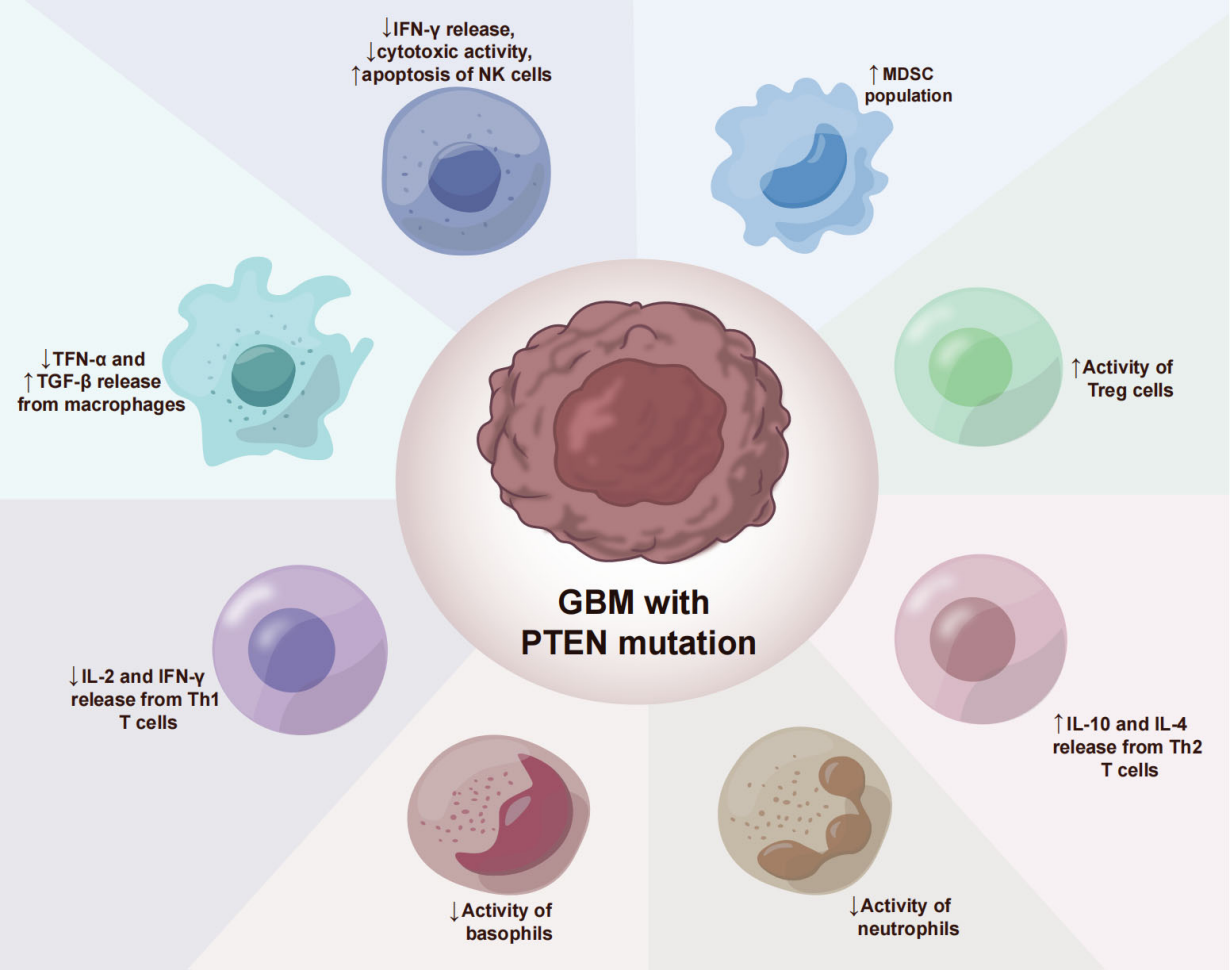
PTEN deficiency immunosuppressive mechanisms in GBM.

The glioblastoma microenvironment (TME) is composed of tumor cells, extracellular matrix (ECM), blood vessels, innate immune cells (monocytes, macrophages, mast cells, microglia, and neutrophils), T cells and neurons, astrocytes, and oligodendrocytes (**Figure 2**). Infiltrating immune cells in GBM are mainly composed of tumor-associated macrophages (TAMs), myelo-derived suppressor cells (MDSC), and T lymphocytes (**Table 2**) (59). A growing number of studies have shown that the tumor immune microenvironment (TIME) plays a crucial role in regulating the growth and metastasis of GBM. Moreover, PTEN participates in the regulation of immune cell signaling; in contrast, PTEN deficiency can lead to an immunosuppressive tumor microenvironment (60) and hinder the anti-tumor immune response. For example, previous studies revealed that the loss of PTEN is significantly associated with reduced T-cell infiltration at the tumor site and resistance to PD-1 blocking therapy (61–64). The loss of PTEN also promotes the accumulation of inhibitory immune cells, such as MDSCs and Tregs, as well as the formation of an immunosuppressive microenvironment during tumorigenation and development (65–67).

**Table 2:** The role of PTEN in regulating signaling proteins in immune cells.

Immune cells	Proteins	Relationship with PTEN	References	Clinical trials
TAMs	IRF1	PTEN deficiency can activate the PI3K-AKT pathway, and IRF1 is up-regulated to promote the secretion of Gal-9, which in turn activates Tim-3 receptor on macrophages, resulting in macrophage enrichment.	(50)	Peng, Guang et al. Oncoimmunology vol. 12,1 2173422. 6 Feb. 2023
	LOX	The loss of PTEN causes the macrophage chemoattractant LOX to be upregulated in a YAP-1 dependent manner.	(51)	Gondek, Tomasz et al. BioMed research international vol. 2014 (2014): 102478.
	Arginase I	PTEN deficiency regulates macrophage activation by activating the PI3K signaling pathway to increase the release of arginase I	(52)	Lorentzen, Cathrine Lund et al. Frontiers in immunology vol. 13 1023023. 17 Oct. 2022,
T lymphocyte	CD44	PTEN mutation induces CD44 overexpression and decreases the number of T cells	(12)	Pazhohan, Azar et al. The Journal of steroid biochemistry and molecular biology vol. 178 (2018): 150-158.
CD8+T	IFN	PTEN regulated the type I interferon pathway via PI3K-independent way	(13, 53)	Boucher, Yves et al. Clinical cancer research: an official journal of the American Association for Cancer Research vol. 29,8 (2023): 1605-1619.
Tregs	Foxp3	PTEN directly regulated the expression of Foxp3, and promoted the Tregs generation and immunosuppressive abilities	(23)	Revenko, Alexey et al. Journal for immunotherapy of cancer vol. 10,4 (2022): e003892.
	mTORC2	PTEN deficiency modulates mTORC2-Akt activity and maintains Treg stability	(54)	Banerjee, Susana et al. JAMA oncology vol. 9,5 (2023): 675-682.
MDSCs	arginase	PTEN deficiency up-regulates arginase activity by activating PI3K signaling pathway, promotes the release of MDSCs, and inhibits T cell function.	(55)	Okła, Karolina et al. Frontiers in immunology vol. 10 691. 3 Apr. 2019
	GM-CSF	PTEN activates the STAT3 signaling pathway, which promotes GM-CSF to up-regulate IL-4Rα on MDSCs, and then mediates IL-13-induced arginase production, thereby inhibiting T cell function.	(56, 57)	Mody, Rajen et al. Journal of clinical oncology: vol. 38,19 (2020): 2160-2169.
	TGF-β1	PTEN activates the Akt pathway to regulate the expression of miR-494 in MDSCs induced by TGF-β1, which promotes the formation of bone marrow mesenchymal stem cells	(58)	Chen, Gang et al. Journal of experimental & clinical cancer research: CR vol. 40,1 218. 30 Jun. 2021,
	IL-6, VEGF, PGE-2	PTEN activates PI3K/AKT/mTOR or STAT3 signaling pathway, and increases the release of factors related to MDSCs proliferation (IL-6, VEGF, PGE-2)	(56, 57)	Bennouna, Jaafar et al. The Lancet. Oncology vol. 14,1 (2013): 29-37.

### 3.1 Tumor-associated macrophages

In the glioblastoma microenvironment, tumor-associated macrophages are the most common infiltrating immune cells, accounting for 40% of the total tumor cells (68). Macrophages constitute the most prevalent non-tumor cells in GBM (23). GiomettoB also found that TAMs can be detected in 100% of GBM cases (69). Two different sources of tumor-associated macrophages have been reported in human glioma, namely from embryonic yolk sac monocytes (70) and from peripheral bone marrow-derived monocytes (50). The immunosuppressive anticancer microenvironment is maintained through the recruitment of monocytes, which are converted into macrophages in the glioma environment. TAMs can be divided into two types, M1 type and M2 type. M1-type TAMs typically express high levels of pro-inflammatory factors, promoting Th1 response and strong tumor-killing ability. In contrast, M2 TAMs promote tissue remodeling and tumor progression and secrete inhibitory inflammatory factors (51). Moreover, glioblastoma-associated macrophages have been reported to exert immunosuppressive effects (52). Previous studies have demonstrated that TAMs in the GBM microenvironment primarily adopt the M2-type polarization (53, 68), which fills the glioma microenvironment and controls tumor progression and immune escape mechanism. The M2 phenotype induces differential expression of receptors, cytokines, and chemokines, which produce IL-10, IL-1, and IL-6, thereby stimulating tumorigenesis and negatively affecting prognosis (54). M2 macrophages stimulate the proliferation and invasion of glioma cells and support the immune escape mechanism (71–73). Giotta’s study confirmed (74) the prevalence of PTEN gene mutation in GBM, which is closely associated with poor prognosis and ultra-low survival rate. A recently published report on GBM showed (75, 76) that PTEN deficiency is associated with high macrophage density. Additionally, PTEN-deficient gliomas can recruit a large number of macrophages in the glioma microenvironment. Another study by Ni et al. (38) revealed that the ability of PTEN-deficient gliomas to induce M2 polarization in macrophages was significantly stronger than that of PTEN wild-type gliomas. In PTEN-deficient glioma cells, the activated AKT pathway inactivates GSK-3β by promoting Ser9 phosphorylation, thereby reducing GSK-3β-mediated degradation of IRF1, leading to the up-regulation of the transcription factor IRF1, which enters the nucleus to promote LGALS-9 gene transcription and Gal-9 expression. The activation of the Tim-3 receptor on macrophages by the Gal-9 ligand, in turn, activates transcription factors associated with M2-type polarization and induces macrophage migration, activation, and enrichment of macrophage-associated angiogenesis pathways in PTEN-null gliomas. Gal-9/Tim-3 is a promising target for the treatment of PTEN-deficient gliomas. Blocking Gal-9/Tim-3 can inhibit the malignant progression of gliomas by inhibiting the M2 polarization of macrophages. A new study on the effect of PTEN deletion on glioblastoma demonstrated (71) increased infiltration of macrophages via the YES-associated protein 1-Lysyl oxidase b1(LOX-b1) -integrin-PYK2 axis. Furthermore, LOX expression was found to activate specific pathways in macrophages, facilitating the recruitment of macrophages to the TME. In the GBM model of PTEN deficiency (77), the loss of PTEN leads to the up-regulation of the macrophage chemotactic LOX in a YAP-1-dependent manner. In circulating monocytes, LOX-dependent up-regulation of β1 integrin receptor signaling drives its penetration into GBM tissues to obtain tumor-associated macrophage phenotype and promotes GBM survival and angiogenesis by secreting SPP1. Interfering with these interactions by inhibiting LOX signals can reduce TAM invasion and inhibit tumor growth. Other studies have found that PTEN regulates the activation of macrophages by activating the PI3K signaling pathway to increase the release of arginase I (78), resulting in a low-inflammation environment. Therefore, arginase I is also a potential therapeutic target.

### 3.2 T lymphocytes

GBM with PTEN mutation shows a reduced number of T cells (17). PTEN mutation can induce an immunosuppressive tumor microenvironment, which is not derived from traditional Treg cells but from tumor cells overexpressing CD44. Other studies have discovered (79) that PTEN regulates the type I interferon pathway in a PI3K-independent manner, inhibits the release of inflammatory factors, and reduces the number of CD8+T cells in GBM. Studies have shown (80) that PTEN lacks the upregulation of mTORC2-Akt activity, and loss of this activity can restore the function of Treg lacking in PTEN. From a mechanism perspective, PTEN can maintain the stability of Treg. Meanwhile, the phosphatase PTEN links Treg stability with inhibition of TH1 and follicular T-helper cell (TFH) responses. Further studies on glioblastoma (18) have revealed that anti-inflammatory cytokine release and T cell activity are significantly reduced in the absence of PTEN and dysregulation of PI3K signaling. Moreover, PTEN inducers or PI3K inhibitors may improve T cell function. Giotta’s study (74) suggested that PTEN mutations were prevalent in GBM, regulating Foxp3 expression and promoting the production of Tregs. Tregs down-regulate T cell activity and regulate innate and adaptive responses to autoantigens, allergens, and infectious agents (81–84). PTEN-deficient tumors usually exhibit a high density of Treg cells in the TME, and Tregs inhibit the function of CD4+, CD8+, and NK cells, and exert immunosuppressive effects in the TME (55, 85, 86).

In addition, T lymphocytes are down-regulated and also exhibit impaired killing function, which is related to TAMs (56). Prostaglandin E2 was found to be produced in the GBM microenvironment, further inhibiting T-cell activity by TAMs and inducing apoptosis. In addition, glioma cells can down-regulate the expression of MHC Class II molecules in microglia and induce ineffective cloning of T cells (87). However, YangI et al. reported that GBM had higher CD8+T cell infiltration compared with pilocytic astrocytoma (57). This differential expression suggests that glioblastoma has a more obvious effect on the local immune microenvironment, but the number does not necessarily represent the potency of the killer cell function. Previous studies have shown that in addition to functional downregulation, CD8+T cells in the GBM microenvironment are involved in the immune escape mechanism.

### 3.3 Medullary inhibitory cells MDSC

Vidotto’s study (81) reported that PTEN deficiency induces an increase in the density of tumor-infiltrating MDSC in TME. MDSCs are a heterogeneous population composed of a large number of immature bone marrow precursor cells, which are activated under pathological conditions and show strong immunosuppressive activity (88). MDSCs protect tumor cells from host immune attack by negatively regulating immune response, including the depletion of amino acids required by T cells such as arginine and cysteine, the generation of reactive oxygen species nitric oxide and peroxynitrite, direct inhibition of macrophages and natural killer cells, and promotion of tumor angiogenesis (58).

In GBM, MDSCs account for a large proportion of tumor immune cells and play an essential role in promoting tumor growth, tumor cell survival, migration, and immune suppression (89). The glioma microenvironment contributes to the immunosuppressive function of MDSCs (90, 91). MDSCs promote glioma growth, invasion, and angiogenesis as well as the proliferation of Tregs cells (92). GIELEN et al. (93) confirmed that the increase of MDSCs in GBM is related to the increase of arginase activity and that the immunosuppressive function was mediated by inhibiting T cells. Studies have found that glioma cells express many factors related to the proliferation of MDSCs (IL-6, IL-10, VEGF, PGE-2, GM-CSF, and TGF-β2); however, blocking the chemokine CCL2 signaling pathway in glioma cells effectively reduces the recruitment of MDSCs (94). Relevant research data revealed a high proportion of microglial cells/macrophages (GAMs) and MDSCs in malignant GBM, with both GAMs and MDSCs having the ability to recruit Tregs to the tumor, further inhibiting the tumor immune response (59, 95). Studies have found that multiple miRNAs in the tumor microenvironment promote the expansion and immunosuppression of MDSCs by targeting inhibiting PTEN and activating the PI3K/AKT/mTOR or STAT3 signaling pathways (96, 97). In addition, GM-CSF up-regulates IL-4Rα on MDSCs via signal transduction and the transcriptional activator STAT3, thereby mediating IL-13-induced arginase production and inhibiting T cell function.

## 4 Glioma immunotherapy targeting PTEN

(1) Evidence suggests that PTEN deficiency plays a crucial role in the development of immunosuppressive cancer phenotypes in glioblastoma and is involved in tumor immune responses. Furthermore, PTEN deficiency activates the phosphatidylinositol 3-kinase (PI3K-Akt) pathway to form an immunosuppressive microenvironment. Since restoring PTEN’s function is currently not feasible, suppressing PI3K signaling represents a potential approach to mitigate PTEN loss (98). Another study showed (17) that the combination of PI3K inhibitor and PD-1 blocker exerts a synergistic effect in PTEN-deficient tumors and can improve the prognosis of patients. In primary cultures of PTEN-deficient gliomas, inhibition of components of the PI3K-AKT-mTOR network resulted in reduced T cell death (99) and enhanced immune response.

(2) PTEN can regulate autophagy and affect GBM immune response through the PI3K/Akt/mTOR mediated signaling pathway and new mTOR independent signaling pathway. Therefore, the inducers of autophagy (PTEN inducers) and the molecular components of the proteolytic system associated with autophagy could be new therapeutic directions for GBM. In addition, some studies have found (37) that proteasome inhibitors specifically induce cell death in GBM organoids with PTEN defects and inhibit tumor growth in mice. Proteasome inhibitors can be used as targeted therapies for GBM.

(3) PTEN mediates immune responses independently of PI3K, so future therapies could also target other downstream pathways and signaling molecules that directly control the immune response in the microenvironment of glioblastoma. For example, PTEN-deficient glioblastomas overexpress CD44 cell-surface adhesion receptors and have a tighter tumor cell phenotype than wild-type glioblastomas (100), which can exclude angioforming and immune cells in TME, making them less responsive to immune checkpoint inhibitors (ICI) (17).

(4) From the above presentation of tumor-associated macrophages in glioblastoma with PTEN deletion or mutation, PTEN deletion or mutation was shown to lead to enhanced aggregation of macrophages into the tumor microenvironment (TME). These findings suggest that targeting M2-type TAMs may be particularly effective against gliomas with PTEN deletion. Inhibition of macrophage M2 polarization by targeting Gal-9/Tim-3 represents a potential target for precise immunotherapy for PTEN-deficient gliomas (38).

Immunotherapy is a therapeutic approach to achieve anti-tumor effects through the action of antibodies on the corresponding receptors. Currently, immunotherapy for gliomas includes vaccine therapy, immune checkpoint therapy, chimeric antigen receptor T-cell immunotherapy (CAR-T), natural killer (NK) cell therapy, and lysosomal viral therapy. However, some problems need to be solved. The main problem with immunotherapy is that normal tissues often have antigenic epitopes identical to those of tumor cells, and activation of the immune response can lead to cross-reactivity between the tumor and the body, resulting in toxicity and autoimmune disease (101). another key challenge is whether immunotherapeutic strategies can overcome the multiple mechanisms of immune evasion in gliomas and generate tumor-specific immune responses (102).In addition, the production of immunotherapeutic vaccines is often complex, with multiple methods of constructing the same vaccine, but the effects of the vaccine will vary (103), and the future of immunotherapy will not be limited to single-pharmacological treatments, but will require a combination of therapies to achieve a broad and long-lasting clinical benefit (101).

## 5 Conclusions and future prospects

A large number of studies have supported the role of PTEN in immune cells and illustrated the immunomodulatory effects of PTEN on glioblastoma TME. PTEN inhibits CD4+/CD8+T cells and dendritic cells while favoring M2 macrophages, Tregs, and MDSCs, participating in glioblastoma progression, metastasis, and immunity. This study outlines the function of PTEN in glioblastoma TME immune cells, as well as their cascade gene activation and clinical outcomes. Increasing evidence demonstrates that targeting PTEN can not only improve the anti-tumor immune function of TME but also enhance the immunotherapy effect, highlighting PTEN as a promising therapeutic target. Nevertheless, whether the recovery of functional PTEN can regulate TME in tumors and improve the sensitivity of tumors to ICB therapy requires further research. Investigating the effectiveness of recovering functional PTEN as a means of cancer treatment holds important clinical significance.
